# *Lactobacillus crispatus* BC5 Interferes With *Chlamydia trachomatis* Infectivity Through Integrin Modulation in Cervical Cells

**DOI:** 10.3389/fmicb.2018.02630

**Published:** 2018-11-06

**Authors:** Carola Parolin, Giulia Frisco, Claudio Foschi, Barbara Giordani, Melissa Salvo, Beatrice Vitali, Antonella Marangoni, Natalia Calonghi

**Affiliations:** ^1^Department of Pharmacy and Biotechnology, University of Bologna, Bologna, Italy; ^2^Microbiology, Department of Experimental, Diagnostic and Specialty Medicine, University of Bologna, Bologna, Italy

**Keywords:** *Chlamydia trachomatis*, lactobacilli, HeLa cells, integrin, women health, probiotics, STIs

## Abstract

Lactobacilli play a crucial role in maintaining the ecological equilibrium of the vaginal niche, preventing the colonization of exogenous microorganisms. Although many studies have discussed the mechanisms displayed by lactobacilli in counteracting several urogenital pathogens, a few data are available on the interaction between lactobacilli and *Chlamydia trachomatis*. This study aimed to elucidate the molecular bases of the interaction among vaginal lactobacilli, the sexually transmitted pathogen *C. trachomatis* and the epithelial cervical cells. We evaluated the *in vitro* activity of 15 *Lactobacillus* strains, belonging to different species (i.e., *L. crispatus*, *L. gasseri, L. vaginalis*), against *C. trachomatis*. In particular, we evaluated the capability of lactobacilli cells to interfere with *C. trachomatis* infection in HeLa cells, by exclusion assays. Lactobacilli significantly reduced *C. trachomatis* infectivity, being *L. crispatus* the most active species. Although a dose-dependent effect was noticed, a significant antagonistic activity was maintained even at lower doses. As other Gram-positive bacteria (i.e., *Streptococcus agalactiae*, *Enterococcus faecalis*, and *Bacillus subtilis*) failed to interfere with *C. trachomatis* infectivity, *Lactobacillus* activity proved to be specific. The potential mechanism of protection was investigated in *Lactobacillus crispatus* BC5, chosen as the model strain. The incubation of HeLa cell line with BC5 cells induced important modifications in the epithelial plasma membrane, by altering lipid composition and α5 integrin subunit exposure. When α5 integrin subunits were masked by a specific blocking antibody or *ITGA5* gene expression was silenced, *Chlamydia* infection was significantly reduced. It follows that α5 integrin subunit is crucial for the pathogen infection process, and the anti-*Chlamydia* activity can be directly linked to membrane properties modifications in cervical cells. The three Gram-positive bacteria used as controls failed to modify the expression of α5β1 integrin. In conclusion, we identified a potential molecular mechanism at the basis of the protection exerted by *L. crispatus* BC5 against *C. trachomatis*, getting insights into the role of the cervico-vaginal microbiota for the woman’s health.

## Introduction

*Chlamydia trachomatis* (CT) represents the agent of the most common bacterial sexually transmitted infection (STI) worldwide (ECDC, 2015). In women, urogenital CT infections are often asymptomatic, thus remaining unnoticed and untreated. This can lead to complications and sequelae including pelvic inflammatory disease, tubal infertility, and ectopic pregnancy (Price et al., 2013; Menon et al., 2015).

A normal vaginal microbiota, dominated by lactobacilli, is crucial for the prevention of several urogenital and sexually transmitted infections, including *Chlamydia* (Gupta et al., 1998; Spurbeck and Arvidson, 2008; Parolin et al., 2015; Nardini et al., 2016; Foschi et al., 2017; Ñahui Palomino et al., 2017). This aspect is strengthened by the demonstration that in case of bacterial vaginosis, a clinical condition characterized by the depletion of lactobacilli, a higher risk of STI transmission and acquisition is reported (Taha et al., 1998; Martin et al., 1999; Wiesenfeld et al., 2003; Abbai et al., 2015).

The protective role of lactobacilli against urogenital pathogens is exerted through different mechanisms including the production of various antibacterial compounds (lactic acid, hydrogen peroxide, bacteriocins, and biosurfactants), the competitive exclusion for epithelial adhesion and the immunomodulation (Kaewsrichan et al., 2006; Borges et al., 2014; Parolin et al., 2015; Younes et al., 2018). In this context, the use of probiotic lactobacilli for the prevention and treatment of several urinary and vaginal tract infections has been extensively evaluated, with different results depending on the *Lactobacillus* species, the strain origin, the concentrations used and the outcome considered (Barrons and Tassone, 2008; Bolton et al., 2008; Spurbeck and Arvidson, 2011; Vitali et al., 2016).

Until now, only a few studies have focused on the *in vitro* interaction between lactobacilli and CT and many aspects remain to be elucidated (Gong et al., 2014; Mastromarino et al., 2014; Nardini et al., 2016). Considering that CT is an obligate intracellular bacterium, characterized by a unique biphasic developmental cycle alternating between the extracellular infectious ‘elementary body’ (EB) and the intracellular ‘reticulate body’ (RB) (Moulder, 1991), lactobacilli can interfere with CT infectivity acting on the different steps of its cycle.

Previous studies shed light on the metabolic interaction between CT and lactobacilli, mimicking what happens in the acid environment of the vaginal niche (Gong et al., 2014; Nardini et al., 2016), but they did not evaluate the ability of lactobacilli cells to compete and interfere with CT EBs infectivity in epithelial cells. It has also been reported that the interaction of lactobacilli with cervical cells results in changes in the structure/functions of the plasma membrane of epithelial cells, especially at the level of α5β1 integrin exposure (Calonghi et al., 2017). The integrin family of receptors is a major target for bacterial pathogens that colonize human tissues or invade specific cell types (Hoffmann et al., 2011; Hauck et al., 2012). Integrins are heterodimeric transmembrane receptors that mediate cell–cell and cell–extracellular matrix adhesion and, as a result, regulate many aspects of cell behavior. In addition to providing a physical transmembrane link between the extracellular environment and the cytoskeleton, they are capable of transducing bi-directional signals across the cell membrane (Hynes, 2002). In this context, the interaction of chlamydial Ctad1 adhesin with β1 integrin subunit has been proposed as one mechanism for EBs binding, invasion, and signaling during entry into host epithelial cells (Elwell et al., 2016; Stallmann and Hegemann, 2016).

The aim of this study was to identify vaginal *Lactobacillus* strains capable of interfering with the infectious process of CT in cervical cells (HeLa cell line) and to understand the rationale of this interaction. A *L. crispatus* strain was chosen as a model to study the molecular mechanisms underlying the anti-*Chlamydia* activity, with particular reference to the modulation of plasma membrane properties and integrin role in HeLa cell line.

## Materials and Methods

### Bacterial Strains and Culture Conditions

**FIGURE 1:**
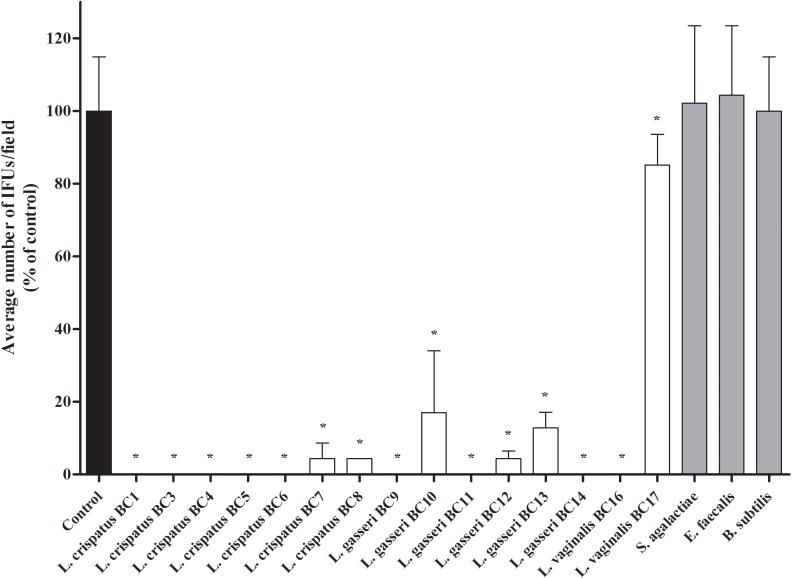
Antagonistic activity of vaginal lactobacilli cells against *Chlamydia trachomatis* infectivity in exclusion assays. Exclusion experiments were performed at the dose of 5 × 10^7^ lactobacilli cells in HeLa cells. *Streptococcus agalactiae*, *Enterococcus faecalis*, and *Bacillus subtilis* were used as reference Gram-positive bacteria. *C. trachomatis* infectivity was evaluated as number of IFUs/microscopic field. Results were expressed in percentage compared with control, taken as 100% (black bar). Bars represent median values, error bars represent median absolute deviations. Statistical significance was calculated vs. control. ^∗^*P* ≤ 0.01.

All the 15 *Lactobacillus* strains included in this study (Figure 1) were previously isolated from vaginal swabs of healthy premenopausal Caucasian women (Parolin et al., 2015). Lactobacilli were grown in de Man, Rogosa and Sharpe (MRS) broth supplemented with 0.05% L-cysteine, for 18 h at 37°C, in anaerobic atmosphere. Anaerobic conditions were achieved by using anaerobic jars supplemented with GazPack EZ (Becton Dickinson and Company, Sparks, MD, United States).

As specificity control, clinical isolates of *Streptococcus agalactiae* and *Enterococcus faecalis* were used. These strains were part of a broad collection including bacteria isolated from vaginal swabs submitted to the Microbiology Laboratory of Sant’Orsola-Malpighi University Hospital of Bologna (Italy) for routine diagnostic procedures. These two strains were grown in Brain Heart Infusion (BHI) broth, in 5% CO_2_ for 18 h at 37°C. *Bacillus subtilis* ATCC 31324 was also included as rod-shaped bacterium control. It was grown aerobically in BHI broth for 18 h at 37°C. All culture media were purchased from Becton Dickinson and Company.

Bacterial cultures were centrifuged at 5,000 × *g* for 10 min at 4°C. Cell pellets were washed and re-suspended in sterile saline to obtain stock cell suspensions of 5 × 10^8^ CFU/mL.

### *C. trachomatis* Propagation and Purification of EBs

In this study, CT strain GO/86, serotype D, was used (Marangoni et al., 2015; Nardini et al., 2016). This strain was clinically isolated from a urethral swab of a patient with non-gonococcal urethritis, submitted to the Microbiology Laboratory of Sant’Orsola-Malpighi Hospital of Bologna, for routine diagnostic procedures and belongs to the laboratory collection. HeLa cells (ATCC CCL-2), a cell line originated from a human cervix carcinoma, were grown to confluent monolayers in 5% CO_2_ at 37°C, inside a Forma Series II 3110 Water-Jacketed CO_2_ Incubator (Thermo Fisher Scientific, Waltham, MA, United States). CT was propagated in HeLa cells, cultured in Dulbecco’s minimal essential medium (DMEM) (EuroClone, Pero, Italy), supplemented with 10% fetal bovine serum, 1% L-glutamine 200 mM, and antibiotics (vancomycin 10 mg/L, gentamicin 10 mg/L, and amphotericin B 0.3 mg/L). For the preparation of EBs, confluent HeLa cells were infected with CT in DMEM medium supplemented with cycloheximide 1 μg/mL, centrifuged at 640 × *g* for 2 h to facilitate cell penetration, then incubated at 37°C with 5% CO_2_ for 48 h (Mastromarino et al., 2014). HeLa cells were then detached and fragmented by sonication, by using a Bandelin sonicator at minimum power. Samples were centrifuged at 500 × *g* for 10 min at 4°C, and supernatants, which contain EBs, were further centrifuged at 40,000 × *g* at 4°C for 1 h. The resulting pellets, containing the purified EBs, were re-suspended in sucrose-phosphate-glutamate (SPG) 0.2 M, divided into small aliquots and stored at −80°C.

### *C. trachomatis* Infectivity Interference Assay

To study the capacity of lactobacilli cells to interfere with the entry of CT EBs into HeLa cells, exclusion experiments were performed (Parolin et al., 2015). HeLa cells were seeded at 2 × 10^4^ cells/cm^2^ in individual tubes containing sterile glass coverslips in 1 mL of medium, and allowed to reach 95–100% of confluence (approximately 1.25 × 10^5^ cells). *Lactobacillus* cells (0.1 mL of the stock cell suspension, corresponding to 5 × 10^7^ CFU) were incubated for 1 h at 37°C with 5% CO_2_ on HeLa cells. Afterward, 5 × 10^3^ CT EBs (0.01 mL of a stock solution of 5 × 10^5^ EBs/mL) were added and further incubated for 1 h [multiplicity of infection (MOI) = 0.04]. HeLa tubes infected with 5 × 10^3^ CT EBs without lactobacilli were used as controls. All the experiments were conducted in triplicate. At the end of the incubation, each HeLa tube was first washed three times with phosphate buffer saline (PBS), then 1 mL of complete medium, supplemented with 1 μg/mL cycloheximide, was added. Individual tubes were centrifuged at 640 × *g* for 2 h to facilitate cell penetration and then incubated at 37°C with 5% CO_2_ for 48 h. CT infection was evaluated by counting chlamydia inclusion forming units (IFUs) by direct immunofluorescence, using a monoclonal antibody against the chlamydial membrane lipopolysaccharide antigen conjugated with fluorescein (Meridian, Cincinnati, OH, United States), as previously reported (Marangoni et al., 2015). Slides were observed under epi-fluorescence microscope (Eclipse E600, Nikon, Tokyo, Japan) equipped with a super high pressure mercury lamp and Plan Fluor DLL 20, 40, 100× lenses. The number of IFUs was counted in 30 randomly chosen 200× microscopic fields. Results were expressed as the percentage (median percentage ± median absolute deviation) of CT infectivity, comparing the number of IFUs of the single experiments with the control tubes.

In order to exclude a potential effect of an acidic environment on CT EBs infectivity, the pH values of the culture medium were measured before the addition of lactobacilli cells and at the end of the incubation.

Exclusion experiments were also performed with *S. agalactiae, E. faecalis*, and *B. subtilis* cells instead of lactobacilli, applying the same conditions described above, to assess the specificity of the anti-CT activity.

*Lactobacillus crispatus* BC4, *L. crispatus* BC5 and *L. gasseri* BC14, selected among the most active strains in counteracting chlamydial infectivity, were used in dose-effect experiments. Specifically, total amounts of 5 × 10^6^ and 5 × 10^5^ lactobacilli cells were tested.

### Nile Red Staining

*Lactobacillus crispatus* BC5 was chosen as a model strain to study the molecular mechanisms underlining the interactions among lactobacilli, CT EBs and HeLa cells plasma membrane. HeLa cells were grown at 70–80% of confluence on glass coverslips, treated with *L. crispatus* BC5, *S. agalactiae, E. faecalis*, or *B. subtilis* cells (5 × 10^7^ CFU) for 1 h at 37°C with 5% CO_2_, and then washed three times with PBS. A stock solution of Nile Red (NR, 9-diethylamino-5H-benzo[alpha]phenoxazine-5-one; Sigma-Aldrich, Milan, Italy) was prepared in DMSO at the concentration of 1 mM and stored protected from light. NR staining was performed for 5 min at 37°C at a final dye concentration of 5 μM. Cells were then fixed with 3% paraformaldehyde for 10 min, and repeatedly washed with 0.1 M glycine/PBS and 1% Bovine Serum Albumin (BSA)/PBS. Specimens were embedded in Mowiol and analyzed by a Nikon Coolscope II equipped with an Eclipse 90i microscope, using sequential laser excitations at 568 nm to reduce spectral bleedthrough artifacts. A Nikon Plan Apo 60× oil objective was used. Fluorescence intensity was quantified on at least eight randomly chosen microscopic fields by using Image J; results were expressed as average fluorescence (A.U.)/field ± SD.

### Fluorescence Anisotropy Measurements

The plasma membrane fluidity of HeLa cells was estimated by means of the fluorescence anisotropy of the hydrophobic probe TMA-DPH (1-4-trimethylammoniumphenil-6-phenil-1,3,5-hexatriene; Thermo Fisher Scientific). HeLa cells (70–80% confluence) were incubated with *L. crispatus* BC5, *S. agalactiae, E. faecalis*, or *B. subtilis* cells (5 × 10^7^ CFU) for 1 h at 37°C with 5% CO_2_, then washed three times with PBS and re-suspended at a final concentration of 3 × 10^5^ cells/mL. The absorbance of the cell suspension was kept lower than 0.15 at the excitation wavelength of TMA-DPH. A few microliters of TMA-DPH stock solution were added to the cell suspension in order to obtain a final probe concentration of 1 μM. Fluorescence anisotropy measurements were performed by using a PTI QuantaMaster fluorimeter (Photon Technology International, North Edison, NJ, United States) equipped with a temperature-controlled cell holder and Polaroid HNP’B polarizers. Temperature was kept at 25°C. Excitation and emission wavelengths were set at 360 and 430 nm, respectively.

### Immunocytochemical Integrin Staining

HeLa cells were grown on glass coverslips at 70–80% of confluence and treated with *L. crispatus* BC5, *S. agalactiae, E. faecalis*, or *B. subtilis* cells (5 × 10^7^ CFU) for 1 h as described above, then washed three times with PBS and fixed in paraformaldehyde. Samples were stained with anti-human CD49e (i.e., anti-human α5 integrin subunit) primary antibody (1:500 in 1% BSA/PBS, BioLegend, San Diego, CA, United States) overnight at 4°C, followed by incubation with Alexa 568-conjugated secondary antibody (1:1000 in 1% BSA/PBS, Thermo Fisher Scientific) for 1 h at room temperature. As a specificity control for α5 integrin subunit signal, untreated Hela cells were also stained with IgG isotype (1:500 in 1% BSA/PBS, Sigma-Aldrich), followed by Alexa 568-conjugated secondary antibody. Specimens were embedded in Mowiol and analyzed in confocal microscopy as described above. Fluorescence intensity was quantified on at least eight randomly chosen microscopic fields by using Image J; results were expressed as average fluorescence (A.U.)/field ± SD.

### Exclusion Assay With Anti – CD49e Antibody

In experiments exploring the role of the α5 integrin subunit in CT infection, HeLa cells grown on glass coverslips at 60% of confluence were first pre-exposed to control (IgG isotype) or anti-CD49e antibody (10 μg/mL) (BioLegend) for 1 h and then incubated with 5 × 10^3^ CT EBs for 48 h at 37°C and 5% CO_2_. At the end of the incubation, samples were extensively washed in PBS, fixed in paraformaldehyde, permeabilized in ethanol and stained with the fluorescein-conjugated chlamydial membrane lipopolysaccharide antibody as described above. Specimens were embedded in Mowiol and analyzed in confocal microscopy as described above, using a Nikon Plan Apo 40× oil objective. Number of IFUs/field were counted by using Image J.

### α5 Integrin Subunit Silencing

siRNA silencing was applied on HeLa cells after 48 h of adhesion, when cells were 60% confluent. The specific siRNA against *ITGA5* gene expression and the scramble siRNA were transfected using lipofectamine according to the manufacturer’s specifications (Thermo Fisher Scientific). Control siRNA or *ITGA5* siRNA (10 nM) were used to treat cells in phenol red-, serum-, and antibiotic-free RPMI. After 48 h of siRNA treatment, cells were infected with 5 × 10^3^ CT EBs for additional 48 h at 37°C and 5% CO_2._ Samples were then washed three times with PBS, fixed in paraformaldehyde, and permeabilized in ethanol. Cells were stained for chlamydial membrane lipopolysaccharide and analyzed by confocal microscopy as described above.

### Western Blot

Control, siRNA, and scramble HeLa cells were washed twice with PBS and lysed for 1 h in lysis buffer (HEPES, pH 7.4 40 mM, glycerophosphate 60 mM, *p*-nitrophenylphosphate 20 mM, Na_3_PO_4_ 0.5 mM NaCl 250 mM, Triton X-100 1%, PMSF 0.5 mM, and 10 mg/mL each of aprotinin, leupeptin, pepstatin and antipain, Sigma-Aldrich) at 0°C. Cell lysates were centrifuged at 12,000 × *g* for 20 min. Supernatants were collected and protein concentration determined by using the Bio-Rad protein assay method (Bio-Rad, Hercules, CA, United States). The proteins were resolved on a 7.5% polyacrylamide gel and immunoblotted with a rabbit anti-human α5 integrin subunit (1:1000 in PBS Tween, Cell Signaling Technology, Danvers, MA, United States), or a rabbit anti- β-actin (1:2000 in PBS Tween, Sigma-Aldrich) antibodies. Detection of immunoreactive bands was performed by using a HRP-conjugated secondary antibody (1:20,000 in PBS Tween, GE Healthcare, Milan, Italy) followed by WESTAR EtaC 2.0 (Cyanagen, Bologna, Italy). Densitometry analysis of immunoreactive bands was done by Fluor-S Max MultiImager (Bio-Rad). Relative quantification of α5 integrin subunit was performed by using β-actin signal as control.

### Statistical Analysis

Infectivity data were analyzed by using R computational software ^1^, applying the non-parametric one-tailed Wilcoxon rank test. Fluorescence intensities were analyzed by Prism software (GraphPad Software, La Jolla, CA, United States), applying the Student’s *t*-test. Differences were deemed significant for *P*-values ≤ 0.01.

## Results

### Interference of Lactobacilli With CT Infection

In the present work we investigated the capability of vaginal lactobacilli to counteract the infection process of *C. trachomatis* in HeLa cells, chosen as an *in vitro* epithelial model of cervical infection. Specifically, we tested the inhibitory activity of 15 *Lactobacillus* strains, previously isolated from vaginal swabs of healthy premenopausal women (Parolin et al., 2015) through an exclusion assay. The effects of lactobacilli cells against *C. trachomatis* infection are reported in Figure 1 and the raw data are listed in Supplementary Table S1. All the *Lactobacillus* strains significantly reduced the chlamydial infection. The most active strains were *L. crispatus* BC1, BC3, BC4, BC5, BC6, *L. gasseri* BC9, BC11, BC14, and *L. vaginalis* BC16.

We excluded that the observed anti-CT activity was related to an acidic environment, since the pH values of the culture media were not modified by the addition and incubation with lactobacilli cells: in particular, pH of DMEM without lactobacilli ranged between 8.0 and 8.1, whereas the pH values at 1 and 2 h post-incubation with lactobacilli ranged between 8.0 and 8.3.

In order to verify if the observed anti-*Chlamydia* activity was restricted to *Lactobacillus* strains, exclusion experiments were also performed with other Gram-positive microorganisms, namely *S. agalactiae*, *E. faecalis*, and *B. subtilis*. No reduction of *Chlamydia* infectivity was noticed for these control strains (Figure 1). In particular, as *B. subtilis* has similar shape and size of lactobacilli, we can suppose that the inhibition exerted by lactobacilli is not due to a physical barrier created by the large rod-shaped bacteria, but to a specific interaction with HeLa cells membrane.

### Dose–Response Effect of Lactobacilli on CT Infectivity

**FIGURE 2:**
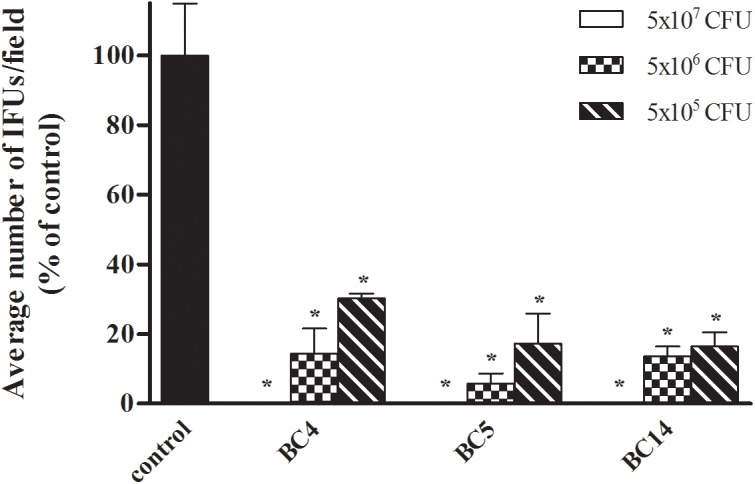
Dose–response effect of *Lactobacillus* cells on *C. trachomatis* infectivity in HeLa cells. Exclusion experiments were performed at different doses (5 × 10^7^, 5 × 10^6^, and 5 × 10^5^ cells) for *L. crispatus* BC4, *L. crispatus* BC5, and *L. gasseri* BC14 strains. *C. trachomatis* infectivity was evaluated as number of IFUs/microscopic field. Results were expressed in percentage compared with control, taken as 100% (black bars). Bars represent median values, error bars represent median absolute deviations. Statistical significance was calculated vs. control. ^∗^*P* ≤ 0.01.

We sought to investigate the effect of different doses of lactobacilli cells on the level of inhibition of CT infection. For this purpose, we selected three strains among the most active ones (*L. crispatus* BC4, *L. crispatus* BC5, and *L. gasseri* BC14) and we evaluated the inhibitory effects of two dilutions, corresponding to the doses of 5 × 10^6^ and 5 × 10^5^ cells (Figure 2). All *Lactobacillus* strains significantly reduced *Chlamydia* infectivity, confirming the strong antagonistic effect of *Lactobacillus* cells toward the infectious process of *Chlamydia*. Despite the antagonist activity was maintained even at lower doses, the level of inhibition was clearly dose dependent for all the lactobacilli.

### *L. crispatus* BC5 Modifies HeLa Cells Plasma Membrane

**FIGURE 3:**
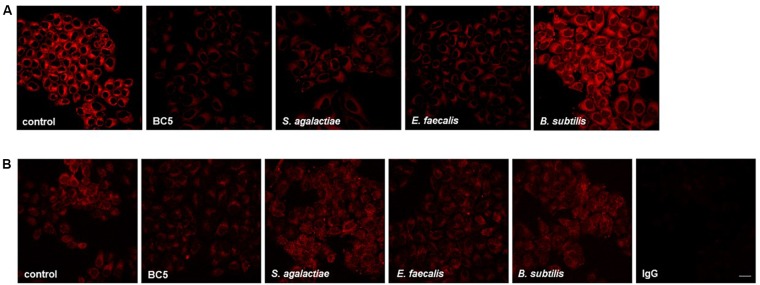
Membrane lipid organization and α5 integrin exposure on HeLa cells incubated with microorganisms. **(A)** HeLa cells were incubated with *L. crispatus* BC5, *S. agalactiae*, *E. faecalis*, or *B. subtilis* for 1 h and then stained with NR. **(B)** HeLa cells were incubated with *L. crispatus* BC5, *S. agalactiae*, *E. faecalis*, *B. subtilis* for 1 h and then stained for α5 integrin subunit. IgG represents specificity staining control. Representative micrographs are shown. Experiments were repeated at least three times with similar results. Bar: 20 μm.

We focused our investigation on *L. crispatus* BC5, chosen as model strain. We thus wondered if 1 h-treatment of Hela cells with *L. crispatus* BC5 cells prior to the infection with CT would be able to induce modifications at the HeLa plasma membrane level, in terms of lipid organization, fluidity, and modulation of protein exposure. HeLa cells were stained with the lipid dye NR and analyzed by confocal microscopy (Figure 3A). The interaction of *L. crispatus* BC5 with HeLa caused a decrease in NR emission fluorescence (Fluorescence intensity A.U. control: 37093 ± 8347, BC5: 16616 ± 6257, *P* = 0.001), indicating a reduced exposure of polar membrane lipids. Steady-state fluorescence anisotropy of TMA-DPH was used to evaluate the possible modifications of the physico-chemical characteristics of HeLa plasma membrane upon interaction with *L. crispatus* BC5. Under our experimental conditions, in the absence of *L. crispatus* BC5, an anisotropy value of 0.266 ± 0.002 was measured. The treatment with *Lactobacillus* cells induced a significant decrease in TMA-DPH anisotropy (0.257 ± 0.001; *P* = 0.006).

It is known that numerous pathogens, including *C. trachomatis*, exploit integrins expressed on target cell plasma membrane in order to mediate adhesion and cell penetration. For this reason, we investigated whether *L. crispatus* BC5 pre-treatment could alter integrin exposure on HeLa plasma membrane, by immunostaining of CD49e and confocal microscopy analysis (Figure 3B). The pre-incubation of HeLa cells with *L. crispatus* BC5 significantly reduced the exposure of α5 integrin subunit on the plasma membrane (Fluorescence intensity A.U. control: 41449 ± 4765, BC5: 27453 ± 3056, *P* = 0.001). Specificity of α5 integrin subunit staining was verified by using IgG isotype followed by fluorescent secondary antibody (Fluorescence intensity A.U. IgG: 3878 ± 337).

In order to verify if the modification of membrane properties are restricted to *L. crispatus* BC5, the same experiments were also performed with other Gram-positive microorganisms. HeLa cells were thus incubated with *S. agalactiae*, *E. faecalis*, and *B. subtilis*. The interaction of *S. agalactiae* and *E. faecalis* with HeLa cells caused a strong decrease in NR emission fluorescence (Figure 3A) (Fluorescence intensity A.U. *S. agalactiae*: 16484 ± 5048, *E. faecalis*: 12911 ± 3413, *P* = 0.001 vs. control, for both comparisons), but these effects on membrane lipid organization were not associated with changes in the membrane exposure of α5 integrin subunit (Figure 3B) (Fluorescence intensity A.U. *S. agalactiae*: 40549 ± 4986, *P* = 0.319; *E. faecalis*: 40059 ± 1333, *P* = 0.214). *B. subtilis* treatment did not affect HeLa plasma membrane lipid organization (Figure 3A) (NR Fluorescence intensity A.U. 42424 ± 7803, *P* = 0.338) nor α5 integrin subunit exposure (Figure 3B) (Fluorescence intensity A.U. 42655 ± 3073, *P* = 0.142). The measurement of anisotropy in Hela cells incubated with *S. agalactiae*, *E. faecalis*, and *B. subtilis* was not performed because these control microorganisms incorporated the TMA-DPH probe.

### Blocking or Silencing of α5 Integrin Subunit Prevent CT Infection

**FIGURE 4:**
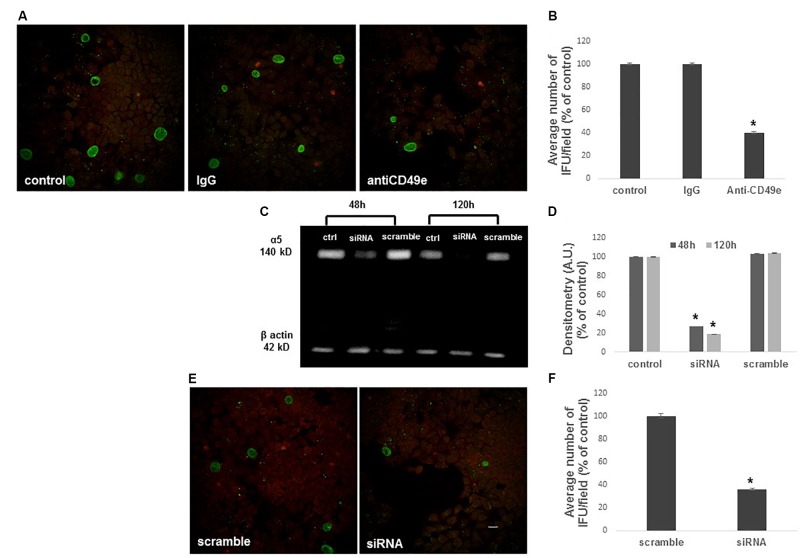
Inhibition of *C. trachomatis* infection by α5 integrin subunit blocking or *ITGA5* gene silencing. HeLa cells were treated or not with an anti-CD49e antibody or control IgG for 1 h, then infected with CT EBs for 48 h. **(A,B)** Specimens were stained for chlamydial membrane lipopolysaccharide antigen. Representative micrographs are shown. *C. trachomatis* infectivity was evaluated as number of IFUs/microscopic field. Results were expressed in percentage compared with control, taken as 100%. Bars represent median values, error bars represent median absolute deviations. Statistical significance was calculated vs. control. ^∗^*P* ≤ 0.01. **(C,D)** Western blotting of α5 integrin subunit expression in control, *ITGA*5 siRNA and scramble Hela cells, evaluated at 48 and 120 h post-siRNA. Quantification of α5 integrin subunit was normalized on β-actin. Bars represent mean values based on three independent experiments, error bars represent standard deviations. **(E,F)** HeLa cells treated with siRNA or scramble were infected with CT EBs for 48 h, and then stained for chlamydial membrane lipopolysaccharide antigen. Bar: 20 μm. Results were expressed in percentage compared with scramble, taken as 100%. Bars represent median values, error bars represent median absolute deviations. Statistical significance was calculated vs. control. ^∗^*P* ≤ 0.01.

To examine the role of α5β1 in CT infectious process in HeLa cells, CT infectivity was evaluated after pre-incubation of HeLa cells with a function-blocking anti-α5 integrin subunit antibody or control IgG. Internalization of CT EBs by HeLa cells was reduced by approximately 60% in the presence of the anti-CD49e antibody (*P* < 0.01), while the pre-incubation with isotype IgG did not interfere with *C. trachomatis* infection (Figures 4A,B).

To further confirm the involvement of α5 integrin in CT infectious process in HeLa cells, we used a specific *ITGA5* siRNA to knockdown endogenous α5 integrin subunit expression. Validated siRNAs were used for the analysis: a specific anti-*ITGA5* and a correspondent negative siRNA were chosen (scramble). A silencing time course was assessed by quantification of α5 integrin subunit protein 48–120 h after silencing with siRNA 10 nM. Total protein lysates were resolved in SDS-PAGE and α5 integrin subunit protein was identified by a specific antibody in western blot. By normalization on the amount of β-actin, quantification of α5 integrin subunit in the specific siRNA sample was compared with the relative scramble sample (Figures 4C,D). Since α5 integrin subunit expression was already greatly reduced after 48 h of siRNA treatment, this time point was chosen as optimal. Following silencing, cells were infected with CT EBs. Transfection of cells with α5 integrin-specific siRNA reduced CT infection by 64%. In contrast, transfection of cells with non-specific control siRNA did not affect CT infection (Figures 4E,F).

## Discussion

The vaginal microbiota of healthy pre-menopausal women is dominated by different species of *Lactobacillus*. They play a fundamental role in the maintenance of microbial homeostasis of the female genital tract, preventing the overgrowth of endogenous opportunistic microorganisms and impeding the colonization of exogenous pathogens (Aroutcheva et al., 2001; Borges et al., 2014). Women with a depletion of vaginal lactobacilli are more likely to be infected by urogenital and sexually transmitted pathogens, such as chlamydia, gonorrhea, and HIV (Gallo et al., 2012; Alcaide et al., 2015).

Although many studies have focused on the mechanisms displayed by lactobacilli in counteracting several urogenital pathogens (Osset et al., 2001; Verdenelli et al., 2014; Breshears et al., 2015; Parolin et al., 2015), up to now only a few data are available about the interaction between lactobacilli and CT. Gong et al. (2014) demonstrated that lactobacilli could inactivate chlamydiae primarily through maintaining an acidic environment in the vaginal lumen by the production of lactic, formic, and acetic acids. Nardini et al. (2016) confirmed the capability of lactobacilli secreted metabolites to strongly inhibit CT EBs infectivity and identified *L. crispatus* as the species with the best anti-*Chlamydia* profile, due to the high production of lactate and the marked consumption of glucose. Moreover, in the same work it has been demonstrated that lactobacilli cells can directly inactivate CT EBs, probably by means of rapid and dynamic modifications of EBs membrane, although to a lesser extent than supernatants. The peculiarity of these previous studies is that the interactions between lactobacilli and CT EBs were simulated in ‘external killing’ experiments, where target epithelial cells were not present. Indeed, in such assays, CT EBs were incubated with lactobacilli supernatants/cells prior to contact with the epithelial cells and residual EBs infectivity was subsequently evaluated. Considering that chlamydiae are obligate intracellular organisms, it is essential to investigate the contemporary interplays of CT, vaginal lactobacilli and host epithelium. For this reason, here we evaluated the capacity of *Lactobacillus* cells to interfere with CT infectivity, placing both microorganisms in contact with Hela cells, which represent the cervical epithelium.

First we found a strong antagonistic activity of *Lactobacillus* cells against the infectious process of CT by means of an exclusion strategy, suggesting that the physiological colonization of the female genital tract by lactobacilli is crucial to prevent the intracellular penetration of the pathogen. Such antagonistic activity is specific for *Lactobacillus* strains, as other Gram-positive microorganisms, i.e., *S. agalactiae*, *E. faecalis* and *B. subtilis*, failed to interfere with CT infectivity process. In this context, the use of probiotic formulations able to create a cervico-vaginal niche rich in ‘health-promoting’ *Lactobacillus* species could be an intriguing approach for the prevention of chlamydial infections and other STIs.

Notably, the present data, integrated with the literature, suggest that vaginal lactobacilli play an important role in countering chlamydial infection exerting concerted and synergetic effects at different stages of the infection process. The metabolites secreted by lactobacilli are able to reduce CT EBs viability during the early infection steps before their internalization into epithelial cells, as previously reported (Nardini et al., 2016). Regarding *Lactobacillus* cells, here we have demonstrated that they act with great effectiveness in preventing the interaction of *Chlamydia* with the eukaryotic cells, by means of exclusion. Although a dose-dependent effect was noticed, a significant antagonistic activity was maintained for all the lactobacilli tested even at lower doses. The fact that low amounts of lactobacilli still exert a significant anti-*Chlamydia* activity prompted us to assume that the prevention of CT infection could be ascribed to modifications in the membrane of epithelial cells, that become less susceptible to CT penetration. Moreover, as previously described for *Candida* spp. (Parolin et al., 2015), we excluded that the most adhesive lactobacilli strains were the most active against CT, strengthening the hypothesis that other mechanisms, besides a steric encumbrance and a saturation of the adhesion sites, should be taken into account.

In the perspective to understand the biochemical bases of the protection exerted by *Lactobacillus*, we further studied the modifications induced by *L. crispatus* BC5 on the host cell membrane, in terms of lipid organization, fluidity, and modulation of protein exposure. We chose *L. crispatus* BC5 as model strain for the following reasons: (i) high inhibitory activity of BC5 toward CT, demonstrated in the present work; (ii) suitability of BC5 to be included in pharmaceutical preparations aimed at the delivery of probiotics to the vaginal mucosa, demonstrated in our previous study (Vitali et al., 2016). Particularly, the interest in a potential application of *L. crispatus* BC5 in pharmaceutical formulations has prompted us to deepen in this strain the knowledge of the molecular mechanisms underlying its antimicrobial action.

It has been previously demonstrated that other vaginal lactobacilli determine strain-specific modifications in the physical properties of HeLa cell plasma membrane. These effects were related to the protective role of lactobacilli against the adhesion of *Candida albicans* on the epithelial cells (Calonghi et al., 2017). Some *Lactobacillus* strains (e.g., *L. crispatus* BC1 and *L. gasseri* BC9) caused a reduced exposure of polar lipids in HeLa plasma membrane with an increase of membrane fluidity. Modification in membrane fluidity could affect receptor binding function by modifying the receptor availability and its rotational and lateral motions. In addition, alterations in lipid fluidity could lead to changes in the tertiary and quaternary structure of the receptor compromising the bacterial ligands recognition (Salyer et al., 1990). Other *Lactobacillus* strains (e.g., *L. gasseri* BC11 and *L. vaginalis* BC15) did not influence HeLa plasma membrane fluidity but they changed α5β1 integrin exposure. Particularly, these strains modified α5β1 integrin organization, leading to the formation of characteristic membrane protein clusters. The consequence of these membrane modifications could be a deviation from receptors native conformation with the resultant inability to correctly recognize and interact with bacterial ligands.

In this work we demonstrated that the incubation of HeLa cells with *L. crispatus* BC5 cells induces important modifications at the level of the epithelial cell plasma membrane, by altering both lipid composition and protein exposure. Upon interaction with lactobacilli, HeLa plasma membrane became more fluid, and reduced the exposure of polar lipids and α5β1 integrin subunits. Notably, when a specific blocking antibody masked α5 integrin subunits or the corresponding gene expression was silenced, CT infection was strongly reduced, supporting the hypothesis that α5 subunit is crucial for CT infection in HeLa cells. This finding, in accordance with Stallmann and Hegemann (2016), confirms the role of integrins as key mediators of the pathogenic process of *C. trachomatis*, and indicates the involvement of the α5β1 dimer in the CT-host cell interaction. However, the adhesion and invasion of CT into epithelial cells rely on other host factors in addition to integrins, such as mannose receptors, ephrin receptor A2 and fibroblast growth factor receptors (FGFR) (Elwell et al., 2016; Lujan et al., 2018). Therefore, it is not surprising that the blocking of α5 integrin subunits or the *ITGA5* gene expression silencing are responsible of only a partial, but substantial, reduction of CT infectivity, rather than a complete abolishment of it.

## Conclusion

Our results allowed us to delineate a mechanism of protection by *L. crispatus* BC5 directly linked to membrane properties modifications in epithelial cells, in particular to the reduction of α5β1 integrin exposure. We are aware that other complementary mechanisms, in addition to the modulation of integrin exposure, could contribute to the ability of lactobacilli to reduce CT infectivity into epithelial cells. Further perspectives will include the study of the modulation of other CT receptors on cervical cells after the interaction with different strains of vaginal lactobacilli.

## Author Contributions

BV, AM, and NC conceived and designed the study, supervised the experimental work, and data analysis. CP, GF, CF, BG, and MS performed the experiments and the statistical analysis. CP, BV, and CF drafted the manuscript. All authors read, reviewed and approved the final manuscript, and contributed in data interpretation.

## Conflict of Interest Statement

The authors declare that the research was conducted in the absence of any commercial or financial relationships that could be construed as a potential conflict of interest.

## Footnotes

1 www.R-project.org
