# Supplementary material for: Lactobacillus crispatus BC5 Interferes With Chlamydia trachomatis Infectivity Through Integrin Modulation in Cervical Cells
Source: Front Microbiol. 2018 Nov 6;9:2630. doi: 10.3389/fmicb.2018.02630 (PMC6232233; doi:10.3389/fmicb.2018.02630)
Supplement: Supplementary file 1 [file Table_1.DOCX]

***Supplementary Material***

**Lactobacillus crispatus BC5 interferes with Chlamydia trachomatis infectivity through integrin modulation in cervical cells**

**Carola Parolin, Giulia Frisco, Claudio Foschi, Barbara Giordani, Melissa Salvo, Beatrice Vitali*, Antonella Marangoni*, Natalia Calonghi**

*** Correspondence:**

Beatrice Vitali: [b.vitali@unibo.it](mailto:b.vitali@unibo.it)

Antonella Marangoni: [antonella.marangoni@unibo.it](mailto:antonella.marangoni@unibo.it)

**Supplementary Table S1.** Raw numbers of *C. trachomatis* IFUs/field detected in exclusion experiments (magnification 200×).

| Control | BC1 | BC3 | BC4 | BC5 | BC6 | BC7 | BC8 | BC9 | BC10 | BC11 | BC12 | BC13 | BC14 | BC16 | BC17 | *S. agalactiae* | *E. faecalis* |  | Control for  *B. subtilis* | *B. subtilis* |
| --- | --- | --- | --- | --- | --- | --- | --- | --- | --- | --- | --- | --- | --- | --- | --- | --- | --- | --- | --- | --- |
| 20 | 1 | 0 | 0 | 0 | 5 | 4 | 5 | 2 | 8 | 0 | 2 | 6 | 1 | 2 | 9 | 44 | 48 |  | 21 | 30 |
| 22 | 0 | 1 | 0 | 0 | 0 | 3 | 4 | 0 | 5 | 0 | 2 | 7 | 0 | 0 | 31 | 18 | 17 |  | 19 | 20 |
| 23 | 0 | 0 | 0 | 0 | 0 | 0 | 1 | 1 | 4 | 4 | 1 | 5 | 4 | 0 | 21 | 18 | 39 |  | 15 | 21 |
| 16 | 0 | 1 | 0 | 0 | 0 | 0 | 1 | 0 | 0 | 0 | 1 | 6 | 0 | 3 | 27 | 16 | 28 |  | 25 | 26 |
| 20 | 1 | 0 | 0 | 0 | 3 | 2 | 1 | 0 | 8 | 1 | 2 | 7 | 0 | 1 | 21 | 12 | 19 |  | 38 | 25 |
| 19 | 0 | 1 | 0 | 0 | 0 | 6 | 3 | 1 | 6 | 2 | 1 | 5 | 3 | 0 | 21 | 28 | 26 |  | 16 | 32 |
| 24 | 1 | 1 | 0 | 0 | 0 | 6 | 1 | 1 | 5 | 0 | 1 | 5 | 0 | 5 | 22 | 17 | 19 |  | 25 | 22 |
| 30 | 0 | 1 | 0 | 0 | 0 | 8 | 1 | 0 | 4 | 3 | 1 | 3 | 2 | 0 | 21 | 19 | 31 |  | 35 | 27 |
| 22 | 0 | 0 | 2 | 0 | 1 | 0 | 3 | 0 | 6 | 0 | 1 | 7 | 1 | 0 | 17 | 28 | 19 |  | 25 | 24 |
| 20 | 1 | 1 | 0 | 0 | 0 | 0 | 1 | 3 | 4 | 0 | 1 | 3 | 0 | 0 | 28 | 11 | 19 |  | 20 | 23 |
| 33 | 1 | 0 | 0 | 0 | 0 | 9 | 1 | 0 | 6 | 1 | 1 | 2 | 0 | 1 | 19 | 35 | 30 |  | 18 | 19 |
| 18 | 0 | 0 | 0 | 0 | 0 | 1 | 2 | 1 | 4 | 0 | 1 | 10 | 1 | 0 | 18 | 31 | 21 |  | 19 | 21 |
| 22 | 1 | 1 | 1 | 0 | 1 | 6 | 3 | 0 | 0 | 0 | 2 | 5 | 0 | 0 | 25 | 44 | 30 |  | 20 | 24 |
| 26 | 0 | 0 | 2 | 0 | 0 | 0 | 2 | 0 | 4 | 1 | 2 | 3 | 2 | 1 | 22 | 12 | 25 |  | 28 | 19 |
| 28 | 0 | 0 | 3 | 0 | 0 | 6 | 1 | 0 | 3 | 0 | 2 | 3 | 0 | 4 | 22 | 18 | 25 |  | 35 | 20 |
| 28 | 0 | 0 | 1 | 1 | 0 | 0 | 0 | 1 | 11 | 1 | 1 | 4 | 4 | 1 | 17 | 31 | 29 |  | 15 | 19 |
| 22 | 0 | 1 | 0 | 0 | 0 | 7 | 1 | 0 | 0 | 0 | 3 | 3 | 0 | 0 | 14 | 26 | 27 |  | 16 | 25 |
| 26 | 0 | 0 | 0 | 0 | 2 | 1 | 1 | 0 | 8 | 3 | 3 | 3 | 0 | 0 | 14 | 28 | 21 |  | 25 | 21 |
| 26 | 1 | 1 | 0 | 0 | 0 | 3 | 1 | 0 | 9 | 0 | 1 | 2 | 1 | 1 | 17 | 39 | 18 |  | 18 | 15 |
| 19 | 0 | 0 | 0 | 0 | 0 | 0 | 1 | 0 | 3 | 0 | 3 | 3 | 1 | 2 | 24 | 23 | 12 |  | 19 | 19 |
| 31 | 0 | 1 | 1 | 0 | 1 | 2 | 4 | 0 | 4 | 1 | 1 | 2 | 0 | 3 | 18 | 30 | 25 |  | 35 | 20 |
| 26 | 0 | 1 | 0 | 0 | 0 | 0 | 2 | 1 | 4 | 1 | 2 | 3 | 2 | 5 | 19 | 24 | 11 |  | 36 | 21 |
| 20 | 0 | 0 | 0 | 0 | 0 | 0 | 1 | 3 | 8 | 0 | 2 | 5 | 0 | 0 | 14 | 27 | 12 |  | 10 | 18 |
| 17 | 0 | 0 | 1 | 0 | 0 | 3 | 3 | 0 | 0 | 4 | 4 | 3 | 1 | 0 | 21 | 22 | 19 |  | 11 | 24 |
| 34 | 0 | 0 | 0 | 0 | 1 | 4 | 1 | 1 | 0 | 0 | 2 | 2 | 0 | 0 | 18 | 30 | 20 |  | 28 | 23 |
| 32 | 0 | 0 | 0 | 0 | 0 | 0 | 2 | 0 | 8 | 0 | 1 | 3 | 1 | 1 | 21 | 19 | 21 |  | 29 | 19 |
| 21 | 0 | 0 | 0 | 0 | 0 | 0 | 1 | 2 | 0 | 1 | 1 | 2 | 1 | 0 | 21 | 28 | 27 |  | 33 | 22 |
| 22 | 0 | 1 | 0 | 0 | 0 | 6 | 1 | 0 | 4 | 0 | 1 | 5 | 2 | 1 | 18 | 10 | 17 |  | 38 | 26 |
| 24 | 1 | 0 | 0 | 0 | 0 | 0 | 2 | 1 | 4 | 3 | 2 | 5 | 1 | 1 | 15 | 28 | 18 |  | 27 | 19 |
| 20 | 0 | 1 | 0 | 0 | 0 | 1 | 2 | 2 | 0 | 1 | 1 | 2 | 0 | 2 | 18 | 29 | 20 |  | 16 | 32 |
| 20 | 1 | 1 | 0 | 0 | 2 | 6 | 2 | 2 | 8 | 2 | 1 | 6 | 0 | 2 | 10 | 17 | 30 |  | 18 | 22 |
| 21 | 2 | 2 | 0 | 1 | 0 | 6 | 3 | 0 | 0 | 0 | 4 | 9 | 0 | 0 | 32 | 25 | 17 |  | 21 | 27 |
| 34 | 0 | 1 | 0 | 0 | 1 | 1 | 1 | 1 | 0 | 0 | 3 | 4 | 0 | 0 | 21 | 26 | 11 |  | 34 | 27 |
| 34 | 2 | 1 | 0 | 0 | 1 | 1 | 5 | 1 | 7 | 4 | 2 | 6 | 2 | 0 | 28 | 31 | 28 |  | 21 | 22 |
| 24 | 1 | 0 | 0 | 0 | 4 | 0 | 1 | 0 | 4 | 1 | 4 | 7 | 0 | 4 | 21 | 19 | 44 |  | 24 | 25 |
| 24 | 0 | 0 | 1 | 0 | 1 | 4 | 3 | 1 | 8 | 0 | 3 | 5 | 0 | 1 | 21 | 22 | 25 |  | 19 | 22 |
| 22 | 0 | 3 | 1 | 0 | 2 | 0 | 1 | 0 | 4 | 2 | 1 | 5 | 4 | 0 | 22 | 29 | 26 |  | 22 | 28 |
| 22 | 1 | 2 | 0 | 0 | 1 | 7 | 1 | 0 | 8 | 1 | 1 | 4 | 0 | 0 | 24 | 31 | 10 |  | 25 | 27 |
| 26 | 0 | 1 | 2 | 0 | 0 | 0 | 4 | 1 | 8 | 0 | 3 | 7 | 0 | 1 | 14 | 17 | 25 |  | 16 | 24 |
| 22 | 1 | 0 | 0 | 0 | 2 | 1 | 1 | 0 | 0 | 0 | 1 | 3 | 0 | 1 | 28 | 22 | 24 |  | 11 | 23 |
| 21 | 0 | 0 | 1 | 0 | 1 | 0 | 1 | 0 | 8 | 0 | 5 | 3 | 1 | 2 | 19 | 24 | 36 |  | 21 | 39 |
| 26 | 0 | 0 | 0 | 0 | 0 | 0 | 2 | 4 | 0 | 0 | 2 | 8 | 1 | 0 | 18 | 22 | 28 |  | 27 | 21 |
| 22 | 0 | 0 | 0 | 0 | 1 | 6 | 1 | 1 | 2 | 1 | 3 | 3 | 0 | 1 | 25 | 23 | 30 |  | 22 | 24 |
| 21 | 4 | 1 | 0 | 0 | 1 | 0 | 3 | 0 | 1 | 0 | 2 | 2 | 3 | 3 | 22 | 18 | 19 |  | 21 | 26 |
| 29 | 2 | 0 | 1 | 0 | 0 | 6 | 1 | 1 | 3 | 1 | 1 | 3 | 0 | 1 | 22 | 30 | 21 |  | 29 | 19 |
| 21 | 2 | 0 | 3 | 0 | 1 | 0 | 2 | 1 | 8 | 4 | 2 | 3 | 0 | 0 | 17 | 19 | 17 |  | 21 | 26 |
| 21 | 4 | 0 | 1 | 0 | 1 | 0 | 1 | 0 | 0 | 5 | 1 | 2 | 2 | 0 | 14 | 22 | 32 |  | 21 | 27 |
| 19 | 0 | 0 | 2 | 1 | 1 | 0 | 1 | 0 | 4 | 0 | 2 | 3 | 0 | 1 | 14 | 24 | 11 |  | 25 | 19 |
| 20 | 2 | 0 | 0 | 0 | 0 | 1 | 0 | 0 | 8 | 3 | 2 | 3 | 5 | 0 | 17 | 27 | 17 |  | 20 | 27 |
| 21 | 4 | 2 | 2 | 1 | 1 | 3 | 1 | 0 | 8 | 3 | 2 | 3 | 1 | 1 | 24 | 28 | 27 |  | 25 | 23 |
| 22 | 3 | 2 | 0 | 0 | 0 | 2 | 1 | 0 | 5 | 2 | 1 | 3 | 0 | 0 | 18 | 27 | 28 |  | 22 | 22 |
| 22 | 0 | 1 | 0 | 0 | 0 | 0 | 1 | 0 | 8 | 0 | 1 | 3 | 2 | 1 | 19 | 26 | 20 |  | 18 | 30 |
| 18 | 1 | 0 | 4 | 0 | 1 | 2 | 2 | 3 | 8 | 1 | 2 | 5 | 0 | 0 | 14 | 17 | 27 |  | 18 | 33 |
| 36 | 4 | 1 | 2 | 1 | 0 | 4 | 3 | 1 | 7 | 1 | 1 | 3 | 0 | 0 | 21 | 32 | 26 |  | 33 | 22 |
| 25 | 2 | 0 | 1 | 1 | 2 | 4 | 1 | 0 | 8 | 0 | 1 | 2 | 0 | 0 | 18 | 19 | 20 |  | 25 | 25 |
| 26 | 2 | 0 | 2 | 1 | 3 | 0 | 2 | 0 | 4 | 2 | 2 | 1 | 1 | 1 | 21 | 20 | 25 |  | 26 | 27 |
| 26 | 3 | 0 | 1 | 0 | 0 | 1 | 1 | 4 | 4 | 1 | 1 | 4 | 4 | 0 | 21 | 21 | 25 |  | 28 | 24 |
| 23 | 2 | 1 | 4 | 0 | 2 | 6 | 1 | 0 | 8 | 0 | 1 | 5 | 1 | 0 | 18 | 39 | 10 |  | 23 | 23 |
| 37 | 2 | 0 | 4 | 1 | 1 | 4 | 2 | 1 | 4 | 0 | 2 | 6 | 3 | 0 | 15 | 21 | 28 |  | 38 | 22 |
| 46 | 4 | 0 | 0 | 0 | 0 | 0 | 2 | 1 | 0 | 0 | 1 | 2 | 1 | 2 | 18 | 21 | 31 |  | 27 | 21 |
| 18 | 0 | 0 | 3 | 0 | 2 | 6 | 2 | 2 | 5 | 3 | 2 | 8 | 0 | 2 | 10 | 29 | 21 |  | 18 | 24 |
| 36 | 0 | 1 | 0 | 0 | 2 | 4 | 3 | 0 | 0 | 2 | 2 | 7 | 0 | 0 | 29 | 18 | 20 |  | 36 | 19 |
| 20 | 1 | 1 | 0 | 0 | 1 | 7 | 1 | 0 | 8 | 0 | 3 | 5 | 0 | 0 | 21 | 21 | 23 |  | 30 | 21 |
| 30 | 3 | 3 | 0 | 0 | 2 | 2 | 1 | 3 | 0 | 0 | 1 | 7 | 0 | 1 | 27 | 25 | 52 |  | 41 | 31 |
| 22 | 2 | 0 | 2 | 1 | 4 | 5 | 1 | 0 | 14 | 0 | 1 | 5 | 1 | 0 | 23 | 25 | 19 |  | 33 | 11 |
| 22 | 0 | 5 | 2 | 0 | 2 | 6 | 3 | 1 | 0 | 1 | 1 | 1 | 1 | 1 | 22 | 39 | 27 |  | 22 | 17 |
| 33 | 0 | 1 | 0 | 0 | 3 | 6 | 1 | 0 | 3 | 1 | 4 | 5 | 0 | 0 | 22 | 30 | 28 |  | 33 | 25 |
| 41 | 2 | 2 | 0 | 0 | 1 | 5 | 0 | 1 | 4 | 4 | 2 | 6 | 0 | 0 | 24 | 32 | 25 |  | 39 | 41 |
| 28 | 1 | 1 | 1 | 0 | 0 | 0 | 3 | 2 | 9 | 1 | 1 | 7 | 0 | 0 | 14 | 48 | 23 |  | 28 | 27 |
| 23 | 0 | 0 | 3 | 0 | 1 | 4 | 1 | 0 | 4 | 5 | 1 | 4 | 0 | 1 | 28 | 22 | 27 |  | 23 | 28 |
| 28 | 0 | 0 | 0 | 0 | 0 | 1 | 1 | 2 | 9 | 0 | 2 | 4 | 2 | 1 | 19 | 22 | 18 |  | 29 | 28 |
| 18 | 2 | 2 | 0 | 0 | 0 | 1 | 2 | 0 | 3 | 0 | 2 | 9 | 1 | 0 | 17 | 20 | 23 |  | 18 | 24 |
| 22 | 1 | 1 | 2 | 0 | 0 | 6 | 3 | 0 | 7 | 0 | 1 | 5 | 0 | 1 | 24 | 15 | 22 |  | 21 | 44 |
| 25 | 2 | 2 | 4 | 0 | 0 | 2 | 2 | 1 | 4 | 0 | 1 | 3 | 0 | 0 | 22 | 18 | 11 |  | 19 | 29 |
| 27 | 0 | 0 | 1 | 0 | 1 | 6 | 1 | 0 | 8 | 1 | 1 | 3 | 5 | 0 | 22 | 21 | 29 |  | 27 | 31 |
| 30 | 3 | 3 | 2 | 0 | 0 | 0 | 4 | 0 | 3 | 0 | 1 | 1 | 0 | 1 | 17 | 19 | 27 |  | 35 | 15 |
| 37 | 0 | 0 | 3 | 0 | 0 | 1 | 1 | 0 | 4 | 2 | 3 | 3 | 0 | 0 | 16 | 46 | 24 |  | 21 | 23 |
| 30 | 1 | 1 | 4 | 0 | 3 | 0 | 1 | 3 | 8 | 1 | 2 | 3 | 0 | 1 | 14 | 12 | 11 |  | 30 | 29 |
| 45 | 0 | 0 | 0 | 0 | 0 | 7 | 2 | 2 | 8 | 0 | 2 | 4 | 1 | 0 | 17 | 21 | 21 |  | 28 | 25 |
| 28 | 0 | 0 | 1 | 0 | 0 | 0 | 0 | 0 | 4 | 4 | 4 | 3 | 1 | 0 | 24 | 20 | 20 |  | 28 | 22 |
| 42 | 1 | 1 | 0 | 0 | 1 | 1 | 1 | 1 | 8 | 0 | 3 | 2 | 0 | 2 | 18 | 12 | 22 |  | 28 | 23 |
| 18 | 1 | 0 | 1 | 0 | 4 | 1 | 0 | 0 | 8 | 0 | 1 | 3 | 0 | 3 | 19 | 35 | 28 |  | 23 | 15 |
| 28 | 0 | 0 | 0 | 0 | 0 | 1 | 2 | 3 | 7 | 0 | 1 | 6 | 0 | 0 | 14 | 31 | 25 |  | 22 | 20 |
| 26 | 0 | 3 | 0 | 1 | 2 | 2 | 3 | 1 | 8 | 0 | 1 | 3 | 1 | 0 | 21 | 27 | 27 |  | 25 | 26 |
| 18 | 1 | 1 | 0 | 0 | 0 | 1 | 1 | 2 | 8 | 2 | 2 | 2 | 0 | 1 | 18 | 25 | 44 |  | 19 | 32 |
| 26 | 0 | 1 | 0 | 0 | 0 | 2 | 2 | 1 | 7 | 0 | 1 | 7 | 1 | 1 | 21 | 38 | 35 |  | 38 | 22 |
| 27 | 0 | 0 | 1 | 0 | 0 | 1 | 1 | 3 | 4 | 3 | 1 | 3 | 0 | 0 | 22 | 27 | 22 |  | 27 | 39 |
| 21 | 0 | 1 | 0 | 0 | 0 | 2 | 1 | 0 | 8 | 0 | 1 | 5 | 1 | 0 | 18 | 44 | 48 |  | 21 | 18 |
| 36 | 3 | 0 | 1 | 0 | 0 | 0 | 5 | 5 | 4 | 0 | 0 | 3 | 2 | 0 | 15 | 23 | 25 |  | 21 | 27 |
| 18 | 0 | 3 | 1 | 0 | 1 | 1 | 1 | 2 | 4 | 2 | 1 | 3 | 1 | 1 | 17 | 51 | 19 |  | 12 | 11 |
